# Multifaceted roles of extracellular RNAs in different diseases

**DOI:** 10.1186/s40779-022-00405-z

**Published:** 2022-08-11

**Authors:** Abdullah Muhammad Sohail, Muhammad Babar Khawar, Ali Afzal, Ali Hassan, Sara Shahzaman, Ahmed Ali

**Affiliations:** 1 Molecular Medicine and Cancer Therapeutics Lab, Department of Zoology, Faculty of Sciences, University of Central Punjab, Lahore, Pakistan; 2 Applied Molecular Biology and Biomedicine Lab, Department of Zoology, University of Narowal, Narowal, Pakistan

**Keywords:** Extracellular RNAs (exRNAs), Extracellular vehicles (EVs), Cancer, Biomarkers, Exosomes

## Abstract

Extracellular RNAs (exRNAs) are novel circulating factors that can be used as biomarkers in various diseases. Their unique and diverse kinds, as well as their role as biomarkers, make them significant biomarkers. There has been immense work carried out since the discovery of exRNAs in circulation and other biological fluids to catalog and determine whether exRNAs may be utilized as indicators for health and illness. In this review, we aim to understand the current state of exRNAs in relation to various diseases and their potential as biomarkers. We will also review current issues and challenges faced in using exRNAs, with clinical and lab trials, that can be used as viable markers for different diseases.

## Background

Extracellular RNAs (exRNAs) are a heterogeneous population of different ribonucleic acids that are found in all biofluids (blood plasma, urine, saliva, etc.). exRNAs are involved in cell differentiation, apoptosis and cell-to-cell communication and regulate various physiological processes, thus modulating gene expression. Previously, exRNAs were thought to be readily degraded by RNases, but recent advances in liquid biopsy have revealed the stability and persistence of exRNAs with the aid of certain other molecules [1, 2]. A decade ago, two important studies revealed that exRNAs exist in conjugation with proteins or lipids, forming complexes and thus enabling the exRNAs to withstand degradation for a longer time [3, 4]. This aspect challenged the intrinsic tendency of exRNAs to escape degradation [5].

The diversity and abundance of exRNAs depend greatly on the sources, such as intracellular compartments, blood and tissues, which makes exRNAs a potential noninvasive biomarker of various diseases. On the diagnostic front, exRNA profiling provides early detection of tumors, hepatic and kidney complications, aging and various other diseases, while on the therapeutic front, exRNAs enclosed in vesicles may offer a safe and precise drug delivery system and well-engineered nanoparticles.

## Exosome biology

Recent research on exRNAs has depicted their dynamic activities that occur and how they communicate information between the cells that play an influential role as signaling molecules. ExRNAs can be divided into two types: coding RNAs, which encode proteins, and noncoding RNAs (ncRNAs), which have various functions, such as assisting the translation of proteins and regulating gene expression. ExRNA protection is largely hidden by the incorporation of membrane-containing vesicles, which are released by cells and are known as potent vehicles of communication. These vesicles are mainly associated with proteins, nucleic acids, and lipids, which perform several biological functions between the parent and recipient cells. Extracellular vehicles (EVs) are mainly classified into microvesicles, exosomes, and apoptotic bodies, which play a crucial role in maintaining homeostasis and in the excretion of unwanted molecular substances [6, 7]. Some microRNAs (miRNAs) are nonexosomal, i.e., they are not associated with exosomes and can be released either by 1) passive release from injured cells or 2) active secretion by protein-mRNA complexes [8]. Messenger RNA (mRNA), which is rarely found among exRNA subpopulations, is usually isolated from certain biological fluids, such as saliva, blood, and urine. Another class of exRNA is miRNAs, owing to their abundance in the cytosol; they regulate various cellular activities, including growth, differentiation and cell death, thus considered as major drivers of cell fate [9–13]. Similarly, exosomal transfer RNAs (tRNA) have shown to be in greater amounts in patients of certain disease as compared to healthy individuals [14]. Nevertheless, the exact mechanism of tRNA fragments is uncertain, but they regulate some pathways and transfer components to other cells to be recognized [15, 16]. In addition, PIWI-interacting RNAs (piRNAs) are one of the largest classes of noncoding RNA (ncRNA) molecules found in the extracellular environment and reside in EVs. However, they are present in meaningful numbers in a few cells and are upregulated in red blood cells compared with plasma [17]. Small ncRNAs (sncRNAs) are more abundant than tRNAs but less abundant than exosomes. The ribonucleoproteins (RNPs), including helicases, polymerases and chaperones, control RNA quality and are involved in the degradation of defective RNAs through surveillance machinery [18–20].

**Fig. 1:**
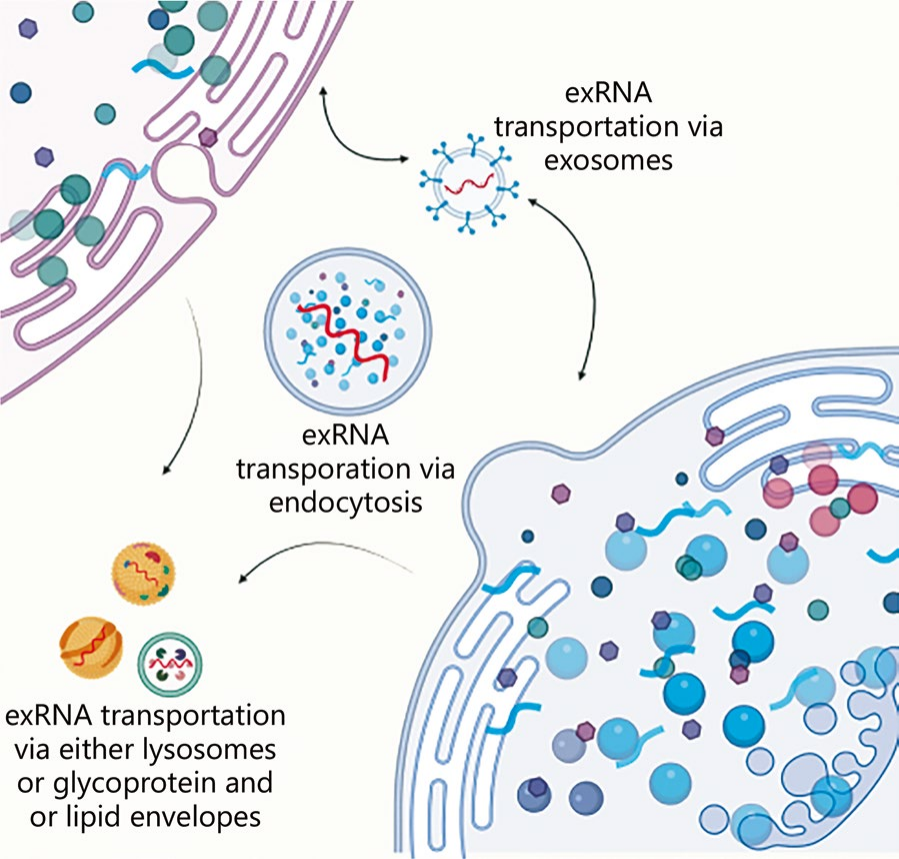
A general view of exRNA biogenesis and its primary function as a transportation cellular entity. exRNA extracellular RNA

What truly defines the characteristics of an exRNA is its origin. Biogenesis is the formation of a cell from another preexisting cell. However, exRNA biogenesis is not limited to a single mode or mechanism. Often, exRNAs are encapsulated in lipoprotein vesicles to evade degradation [1] (Fig. 1). As described previously, various cellular entities are responsible for exRNAs, and exosomes in particular have been linked to RNA as their carriers. One common biogenesis mechanism is endocytosis [21], where two different cells, secreting cells and recipient cells, are responsible for exRNA biogenesis. Endosomes and lysosomes secreted via endocytosis develop into late-stage endosomes and are excreted out of the cell as either exosomes, lipoproteins, or RNPs and then transported toward the recipient cell [1]. Exosomes are analogous to high-density lipoproteins (HDL) and low-density lipoproteins (LDL) in the transportation of exRNAs. For example, HDL transports a specific profile of exRNA from a variety of cell types to recipient cells [22]. Finally, the RNPs are stabilized to carry the exRNA [2], which suggests the possibility of exRNAs existing in more carrier-free forms rather than in association with vesicular bodies. These extracellular vesicular bodies soon enter the recipient cell via cell receptors and other complexes to alter the expression of various genes. These expression profiles drive differences in diseases among individuals, making them novel biomarkers of disease [21]. In 2010, researchers studied the release of HEK293 cell-derived exosomal miRNAs and discovered a dynamically controlled secretory ceramide-dependent mechanism. This mechanism could promote the sorting of endosomes into exocytic multivesicular bodies (MVBs) [23]. Later, another study confirmed the reliance of miRNA secretion into endosomes on ceramide and KRAS mutations [24]. However, there is still much uncertainty about the actual mechanisms involved. In more recent studies, after reaffirming the enigmatic nature of exRNAs, it was observed that many methods of identifying RNA were more related to extracellular complexes [2].

## Extracellular RNA as a diagnostic biomarker

The expeditious use of exRNA in clinical trials is due to its expression in various health conditions; therefore, it has been recognized as a potential biomarker. For example, miRNAs have been studied extensively in the clinical profiles of patients with a variety of diseases, suggesting that they are promising molecular markers for diagnosis and treatment. For instance, miR-21 has been reported in hepatitis, chronic kidney disease (CKD), and brain tumor, in which it has shown significant correlations with other parameters [25–27].

Other types of exRNA have also been reported as molecular markers, such as circulating RNAs (circRNAs), which are highly stable, conserved, and have tissue-specific expression patterns and have been suggested as potential diagnostic and prognostic biomarkers [28]. Multiple sclerosis (MS) currently does not have a single definite diagnostic test, and there are ongoing efforts to discover a diagnostic marker [29]. Fortunately, it is possible to utilize exRNAs as a diagnostic biomarker in MS, as many studies have revealed exRNA associations with diagnostic and therapeutic fields [30].

In 2017, Ebrahimkhani et al. [31] tried to fill the gap when they used a blood-based assay for serum exosomal miRNA detection in a cohort (*n* = 36, patients = 25, and control = 11) of MS patients who were experiencing relapse and remission. They found several miRNAs as biomarkers in the diagnosis of MS. Despite this, many types of cancers are also associated with clinical applications of exRNAs, and they have also been shown to acquire both beneficial and detrimental effects on specific conditions. For example, in a study, 5 exRNAs, including 3 mRNAs (SPINK7, PPL, and SEMA4B) and 2 microRNAs (MIR140-5p and MIR301a), showed downregulation in gastric cancer (GC). These exRNA candidates were isolated from the saliva of GC patients, which shows their ultimate potential utility for the use as noninvasive biomarkers in monitoring GC [32].

In another study, miR-21 showed significant elevations in the serum of glioblastoma patients, and these single exRNAs may target and regulate hundreds of genes [33]. Some particular retrotransposon RNAs originating from tumor cells have shown increased expression compared to normal cells, making them easier to isolate from biofluids. This characteristic makes them a unique and potential biomarker in cancers [34]. To some extent, exRNAs have been found to have a significant role in CKD, but the exact relevance remains unclear. Several changed expression patterns have also been detected in CKD during hemodialysis and after kidney transplantation [35].

A comparative study described a comparison between lupus nephritis (LN) patients and controls through miRNA profiles of kidney biopsy. miRNA microarray chip analysis of renal biopsies revealed differential expression of approximately 66 miRNAs, thus concluding that exRNAs may be an element in the pathogenesis of LN and a potential invasive diagnostic biomarker [36]. A detailed description of the biomarkers of exRNAs in various diseases is given in Table 1.

**Table 1:** Overview of several exRNAs as diagnostic biomarkers in different diseases

Biomarker	Type of disease	Method	Medium	Significance	References
miR-29	Liver cirrhosis	qPCR	Serum	Lower levels show disease progression	[37]
miR-29a	Liver fibrosis	qPCR	Serum	Low levels show advanced liver fibrosis	[38]
miR-122/miR-155	Acute and chronic liver injury	TaqMan MicroRNA assay	Serum and plasma	Upregulated in hepatocytes and a central regulator of inflammation	[39]
miR-30	Muscle injury, muscle disuse atrophy	qRT-PCR	Skeletal muscle	An interesting biomarker of muscle homeostasis and muscle disease	[40]
miR-223	Acute liver failure and liver cirrhosis	qPCR	Tissue and serum	Upregulation restricted to hepatocytes, also showed significantly higher levels in serum	[41]
miR-21 amplification	Glioblastoma	RT-PCR, microarray, Western blotting, immunohistochemical analysis	Cell lines, CSF	Regulator of EGFR expression, cell-cycle and signaling pathways	[26, 42]
miR-603/miR-181d ratio		RT-PCR, Western blotting	Tissue, cell line	Coregulators of MGMT expression	[26]
EGFR amplification		RT-PCR, Western blotting	Tissue	Enhanced cell survival and proliferation via EGFR-PI3K pathway	[26]
miR140-5p/miR301a	GC	RT-qPCR	Saliva	Downregulated in the GC	[32]
miR-26a/b	Oral squamous cell carcinoma	PCR	Epithelial tissue	Downregulation and function as a tumor suppressor	[43]
miR‐34a	Brain aging	qPCR	Blood and Plasma	Accessible biomarkers for age‐dependent changes in the brain	[44]
miR-24-3p	Aging	RT-qPCR array, RT-qPCR validation	Saliva	Nonspecific screening biomarker for aging	[45]
miR-29a/miR-29b	GDM	PCR	Serum	Downregulated in the GDM	[46]
miR-150/miR-192/miR-27a	Diabetes mellitus	Microarray profiling	Blood	Correlation between raised levels of fasting glucose and altered levels of miR-27a and miR-320a	[47]
miR-9/miR-29a	T2DM	qPCR	Serum	Deregulated in T2DM	[48]
miR-155/miR-181a	T1DM	Microarray profiling and qPCR	Serum	Deregulated in T1DM	[49]
miR-21	Type 1 autoimmune hepatitis	Real-time qPCR	Serum	Correlation with the histological grades of inflammation	[27, 50]
miRNA-let-7a/miRNA-92a/miRNA-648a	Multiple sclerosis	qPCR	Plasma	Significantly lower expression	[51]
miR-15b/miR-34a/ miR-636	Diabetic kidney disease	qRT-PCR	Urine	Upregulated in urine pellets	[52]
miR-21-5p	Diabetic kidney disease	qPCR	Urine	Upregulation and associated with pathogenesis of renal dysfunction	[53]
miR-21	Renal fibrosis in diabetic nephropathy	qRT-PCR	Renal cortical tissue	Targets known fibrotic signaling proteins	[25]

*GC* gastric cancer, *GDM* gestational diabetes mellitus, *T2DM* type 2 diabetes mellitus, *T1DM* type 1 diabetes mellitus, *CSF* cerebrospinal fluid, *EGFR* epidermal growth factor receptor, *GFR* glomerular filtration rate, *PCR* polymerase chain reaction, *qPCR* quantitative PCR,* qRT-PCR* quantitative reverse transcription polymerase chain reaction, *RT-qPCR* reverse transcription quantitative PCR, *MGMT* O^6^-methylguanine methyl transferase

### Extracellular RNAs in cancer

Extracellular RNAs have been shown to be potential candidates in cancer diagnosis and prognosis because they are highly specific and highly sensitive indicators, reflecting the dynamics of the cells more accurately than DNA [54]. The noninvasive nature of exRNA biomarkers makes them more reliable candidates in the diagnostics of several cancers. For example, in the clinical diagnosis of GC, different expression levels of extracellular vesicle-derived exRNAs have been detected. Most of these exRNA biomarkers can even enhance the ability to distinguish a benign or malignant tumor [55]. Tumor cells release more vesicles than normal cells, which ultimately affects the tumor microenvironment, thus promoting tumor growth. For instance, long noncoding RNAs (lncRNAs) and miRNAs released by hepatocellular carcinoma cells into surrounding cells altered normal functioning and promoted the multifocal progression of tumors [56].

A number of exRNA biomarkers along with their expression abundance have been described. The high-throughput RNA sequencing and microarray analysis of these biomarkers can help researchers to collect extensive and valuable data about several cancers [57]. Previously, a number of studies have suggested that exRNAs loaded in vesicles are released into the circulation by tumor cells to communicate with cells in close proximity as well as with distant cells [58]. These vesicles play a promoting role in primary breast cancer development, invasion and metastasis. Prostate cancer is another prevalent malignancy that affects the male reproductive system. While examining cancerous prostate tissues, differential expression of reactive oxygen species, the p53 pathway, oncogenes and tumor suppressor genes in tumor vesicles was found [59]. Furthermore, urine contains biomarkers for malignancies of the reproductive system. PCA3, for example, is a lncRNA that is expressed and found in considerable amounts in patients with prostate cancer [60]. A comprehensive study found various exRNA signatures with significant potential for prostate cancer diagnosis [61]. Moreover, the study of some other exRNAs, such as miRNAs and piRNAs, has revealed complete extracellular ncRNA data in human saliva for advanced research on biomarkers [62]. Various oncogenic and oncosuppressor miRNAs, including miR-21, miR-223, miR-378e, miR-143 and miR-10b, have been reported to increase the invasion of tumors in a variety of cancers [63]. Last, a high correlation was observed among 7 differentially expressed genes and lung cancer through RNA sequencing technology [64]. Despite the fact that a number of exRNA biomarkers have been discovered for cancer diagnosis, a systematic identification of these novel biomarkers can provide accurate knowledge about the populations of EV-associated exRNAs and the analyses of their cargo.

### Role of extracellular RNA in liver disease

Extracellular RNAs are vital molecular entities in the pathogenesis of various diseases, including liver cirrhosis, liver fibrosis, and chronic hepatitis.

Disease progression, as well as the healing process through regeneration, is regulated by various types of exRNAs (especially miRNAs), most of which have been distinguished as biomarkers for liver diseases. Hepatic stellate cells (HSCs) during liver fibrosis differentiate into transitional cells called myofibroblasts, which promote extracellular milieu release. These activated HSCs start to produce cellular communication network factor 2 (CCN2), which is related to the underexpression of miR-214, showing its regulatory effect on CCN2 [65]. Furthermore, miR-199a-5p targets the CCN2 3′-UTR and inhibits the production of CCN2; when they are transported to activated HSCs, they inhibit CCN2 3′-UTR activity [66]. This increases the extracellular material, thus causing liver fibrosis.

Hepatocellular carcinoma may also develop as a consequence of liver cirrhosis, making it difficult to diagnose the disease. An extensive investigation of proteomics through the serum of liver cancer patients, liver cirrhosis patients and control patients verified a hypothesis differentiating liver cancer and liver cirrhosis [67]. miRNA-451a was shown to be differentially expressed in the serum of 25 liver cirrhosis patients with early-stage liver cancer in comparison with 74 cirrhotic patients without liver cancer [68]. Nonalcoholic steatohepatitis (NASH) can lead to liver fibrosis; for example, miR-122 is an early biomarker for liver injury due to NASH. The upregulation of miR-122 was compared with serum alanine aminotransferase (ALT) [69]. A previous study revealed that the downregulation of miR-122 promotes alterations in lipid metabolism [70], as exRNAs usually form lipoprotein-RNA complexes [71]. However, the mechanism of lipid homeostasis under the effect of miR-122 has yet to be defined further.

Nonalcoholic fatty liver disease (NAFLD) can progressively lead to NASH. A combined analysis of four serum exRNAs (miR-21-5p, miR-151a-3p, miR-192-5p, and miR-4449) showed satisfactory diagnostic potential for NASH in NAFLD [72]. The plasma levels of novel tRNA-derived fragments predicted liver fibrogenesis risk in NAFLD [73]. miR-20a and miR-27a are useful biomarkers, as they have shown enhanced downregulation upon the downregulation of miR-126. The two previously mentioned exRNAs are significantly associated with the severity of NAFLD. On the other hand, miR-122 aids in the amplification of viral translation in hepatitis C by changing the structure of the internal ribosomal entry site [74]. miR-802 showed the same results on viral replication in hepatitis B [75]. Viral RNAs of heterogeneous lengths have also been observed circulating in blood as capsid-antibody complexes in hepatitis B [76].

### Role of extracellular RNA in lung fibrosis

Similar to liver fibrosis, exRNAs also have a versatile role in lung fibrosis (LF). The extracellular matrix (ECM) contains many subcellular entities that accumulate during myofibroblastic transition, resulting in several exRNA entities being released out of the cell. Exosomal miR-1343 plays a role as a potent inhibitor of TGF-β signaling, and it has shown high expression in HL-69 human leukemia cells. This high expression was then transferred to the A549 lung epithelium, where it inhibited TGF-β receptors 1 and 2 [65]. A research study by Yao et al. [77] showed that miR-328 was overexpressed in M2 macrophages and contributed to pulmonary fibroblast proliferation and the development of LF by regulating FAM13A in rats. Another study also showed the impact of let-7d-5p downregulation, which indicates its involvement in pulmonary inflammation by regulating several pathophysiological processes, such as the release of ECM for the development of idiopathic pulmonary fibrosis (IPF). A positive correlation between let-7d-5p and the diffusing capacity of the lungs for carbon monoxide/alveolar volume showed its association with IPF severity [78].

### Role of extracellular RNAs in diabetes and obesity

As discussed earlier, an exRNA can envelop itself into lipids and plasma lipoproteins, which are important distributary agents that can be converted to a different form of lipid, resulting in many lipid-related illnesses [79]. Obesity may also lead to insulin resistance (IR), which causes the body to be inactive in producing insulin. Various diagnostic practices ranging from glucose testing to oral testing are performed to diagnose an individual with obesity [80]. In a recent review, certain exRNAs were shown to function as potential therapeutics for IR [81]. Recently, a study revealed a relationship between the etiology of diabetes and exRNAs. Furthermore, the findings of exRNAs in serum and plasma offer possible targets for the development of novel drugs [82]. In a cohort comparative study, it was found that individuals with diabetes showed higher levels of circulating EVs than control patients. The same study also concluded that patients who developed the disease in 5 years (or more) also had higher levels of EVs [83]. Moreover, studies have aimed to form a library of exRNA in human aging after the detection of short and long RNAs in a single sequencing step [84].

### Role of extracellular RNAs in aging

Aging is an irreversible process that is influenced by numerous factors. In a recent study, the levels of various kinds of exRNAs increased with age [85]. The results offered timely and pertinent information regarding the serum exRNA repertoire and how it changes as people age. The potential diagnostic and therapeutic use of EVs in age-related diseases has sparked renewed interest in these nanosized vesicles [86]. Extracellular vesicles have some unique biological features and play several important physiological roles. miRNAs and their mRNA targets are essential regulators of cellular senescence, and their expression changes with age in circulating peripheral blood mononuclear cells (PBMCs) [87]. Long noncoding RNAs (lncRNAs) are noncoding RNAs (ncRNAs) with transcript lengths greater than 200 nucleotides that play vital roles in the regulation of gene expression. Therefore, unveiling the molecular mechanisms of lncRNAs that underlie senescence may make it easier to diagnose and treat disorders associated with aging [88]. Many future studies will aim to elucidate the complexities of age and its relation to exRNAs. Another study evaluated exRNAs in PBMCs of healthy subjects, and the results showed biological inactivity due to aging. The study also identified key alterations in many aging-associated biological processes through differential expression analysis [89]. Likewise, another study indicated that exRNA and age-related expressions coincided with certain previously known genes, again indicating the potential of exRNAs as biomarkers for aging [90].

## Clinical trials

The use of exRNAs and their potential was first properly taken into account by the NIH Common Fund-supported Extracellular RNA Communication Consortium (ERCC1) in 2013. ERCC1 documented numerous manuscripts and publications. Moreover, they allocated various sources and their correlating applications [91]. Since exRNAs may function as EVs, various studies have shown a positive impact on cancer therapeutics and the use of exRNAs, specifically in lung cancer [92]. Doxorubicin, a drug used in for cancer treatment, has been optimized to be more effective in codelivery with exRNAs [93]. Other diseases, such as heart failure, have also shown an association with exRNA expression. Whether it be the subdual, production, or removal of EVs, these strategies are all notable approaches [94]. Many clinical trials have tried to modify exRNAs to target a specific cell or cell type [95]. Another investigation discussed how EVs support numerous immune responses, such as immunosuppression, and aid in cancer countermeasures [96]. EVs have also shown many similarities to enveloped viruses, which can be used as prototypes to better understand the potential for these vesicles to operate as vectors for RNA delivery [97]. One study concluded that battling cancer with combination therapy has proven to be more effective than conventional monotherapy [98]. When cancer cells were subjected to 8 Gy radiation, CDCP1 was shown to be elevated as an ideal tumor-associated antigen (TAA) in lung cancer cells. These overexpressed TAAs are subsequently transported to dendritic cells by EVs, accelerating CD4^+^ and CD8^+^ T-cell aggregation, infiltration, and tumor killing [99]. Conversely, during the priming of the premetastatic niche, tumor-derived exosomes have been shown to carry molecular signals important in angiogenesis and stromal remodeling for tumor cell adhesion and proliferation [100]. Exosomes hold potential in cancer therapeutics, as evident from various studies; however, further preclinical trials are necessary to consider them a viable approach [101, 102].

## exRNAs as therapeutics

Since exRNAs are thought to be associated with many local biomolecules, including exosomes, exRNAs have a multitude of biological functions and therefore show correlation to diseases [103]. For clinically meaningful treatment routines, strategies for effectively delivering RNA are frequently needed. The use of viral vectors as RNA carriers with a high transfection efficiency has been investigated. Such use of viral vectors, including adeno-associated viruses, lentiviruses, and retroviruses, is limited owing to the risk of insertional mutagenesis and immunogenicity. In a recent study, fetal bovine serum (FBS) and its influence were tested on cell cultures, revealing that current analysis and protocols lack any notable changes [104]. For more reliable and scalable cancer treatment methods, modification of miRNAs that span both tumor cell proliferation and T-cell-mediated antitumor immunity will lead to crucial developments in the near future [105].

In regard to the translational uses of exRNAs, researchers have begun to think outside the box. Stem cell modification of exosomes has suggested the possibility of facilitating the multiplication of cells at a critical site, such as acute kidney injury [106]. Another study concluded that implementing exRNAs as epigenetic modifications could lead to advancements in transplantation and genome-wide studies practices; however, few studies have focused on this topic [107]. Furthermore, a number of studies have documented that miRNA absorption into recipient cells via EVs alters gene expression and physiological activities and that miRNA profiles are altered in patients with various disease types and/or statuses [1].

Directed distribution of exRNA-loaded EVs for gene therapy and treatments targeting exRNAs have been implicated in kidney disease development, which is a potential therapeutic strategy [106]. Recently, a study investigated the effectiveness of cell-free DNA (cfDNA) [108] and exRNAs in cancer cases by identifying viable biomarkers [109].

## Conclusions

After exploring the nature of the circulating marker exRNA, we conclude that its use as a biomarker has opened an entirely new realm of diagnostics in various diseases, ranging from cancer to obesity. Although there is increasing interest in exRNA and EV biology, there is still a critical need for rigorous, hypothesis-driven investigations to develop model systems that allow for a molecular understanding of exRNA. In developing such models, we will come closer to unveiling the full potential of exRNA in medical fields. In the future, alternative unknown carriers for transporting exRNA as well as the importance of molecular function in cell biology can be investigated. Hence, the significance of circulating biomarkers as diagnostic and prognostic indicators of disease will be increased when exRNAs are progressively considered in various studies and cohorts.

## Data Availability

Not applicable.

## Abbreviations

ALT: Alanine aminotransferase
CCN2: Cellular communication network factor 2
CCN2 3′-UTR: Untranslated regions from 3′ of CCN2
circRNAs: Circulating RNAs
CKD: Chronic kidney disease
ECM: Extracellular matrix
ERCC1: Extracellular RNA communication consortium
exRNAs: Extracellular RNAs
EVs: Extracellular vehicles
FBS: Fetal bovine serum
GC: Gastric cancer
GGT: γ-Glutamyltransferase
HDL: High-density lipoproteins
HSCs: Hepatic stellate cells
IPF: Idiopathic pulmonary fibrosis
IR: Insulin resistance
LDL: Low density lipoproteins
LF: Lung fibrosis
LN: Lupus nephritis
lncRNAs: Long noncoding RNAs
miRNA: MicroRNA
MMP2: Matrix metalloproteinase 2
mRNA: Messenger RNA
MS: Multiple sclerosis
MVBs: Multivesicular bodies
NASH: Nonalcoholic steatohepatitis
ncRNAs: Noncoding RNAs
PBMCs: Peripheral blood mononuclear cells
piRNAs: PIWI-interacting RNAs
RNPs: Ribonucleoproteins
sncRNAs: Small noncoding RNAs
TAA: Tumor-associated antigen
TGF-β1: Transforming growth factor-β1
tRNA: Transfer RNA
